# Does Soil Contribute to the Human Gut Microbiome?

**DOI:** 10.3390/microorganisms7090287

**Published:** 2019-08-23

**Authors:** Winfried E.H. Blum, Sophie Zechmeister-Boltenstern, Katharina M. Keiblinger

**Affiliations:** Institute of Soil Research, Department of Forest and Soil Sciences, University of Natural Resources and Life Sciences Vienna (BOKU), Peter Jordan-Straße 82, 1190 Vienna, Austria

**Keywords:** soil biodiversity, human health, land use, lifestyle, gut microbiota, soil microbiology, global change, nutrition, organic-agriculture, urbanization

## Abstract

Soil and the human gut contain approximately the same number of active microorganisms, while human gut microbiome diversity is only 10% that of soil biodiversity and has decreased dramatically with the modern lifestyle. We tracked relationships between the soil microbiome and the human intestinal microbiome. We propose a novel environmental microbiome hypothesis, which implies that a close linkage between the soil microbiome and the human intestinal microbiome has evolved during evolution and is still developing. From hunter-gatherers to an urbanized society, the human gut has lost alpha diversity. Interestingly, beta diversity has increased, meaning that people in urban areas have more differentiated individual microbiomes. On top of little contact with soil and feces, hygienic measures, antibiotics and a low fiber diet of processed food have led to a loss of beneficial microbes. At the same time, loss of soil biodiversity is observed in many rural areas. The increasing use of agrochemicals, low plant biodiversity and rigorous soil management practices have a negative effect on the biodiversity of crop epiphytes and endophytes. These developments concur with an increase in lifestyle diseases related to the human intestinal microbiome. We point out the interference with the microbial cycle of urban human environments versus pre-industrial rural environments. In order to correct these interferences, it may be useful to adopt a different perspective and to consider the human intestinal microbiome as well as the soil/root microbiome as ‘superorganisms’ which, by close contact, replenish each other with inoculants, genes and growth-sustaining molecules.

## 1. Introduction

The large diversity of microbiota in soil affects its microbial ecology, including its primary productivity and nutrient cycling. In addition, soil is part of the habitat of humans, providing space for living, recreation and food production [1]. From early childhood, we are in contact with soil; we taste it, we inhale it, and we drink water which has passed through soil. Moreover, we ingest plants grown on soils together with soil microbiota. Since pre-history, humans have also willingly consumed soils as a supplement to their otherwise nutrient-poor local diet, a habitude called “geophagy”. They have used certain soils as detoxifying agents necessary for making certain food products edible, and for medicinal purposes, usually as treatments for gastrointestinal ailments [2].

Meanwhile, the human microbiome has become a major field of biomedical research, especially the intestinal microbial community, which plays a major role in human health and disease [3]. The intestinal environment is subject to a constant influx of microbial colonizers [4]. However, each individual harbors a distinct microbiome that can be readily differentiated based on his resident microbes. Transiting diet-associated bacteria may contribute to gut metabolic capacities [5]. The microbial community of the gut is very dynamic and consists of autochthonous and allochthonous members (that are absorbed by food and water, as well as by direct contact with the environment/soil in which they live [6,7]).

In view of the functional similarities between the intestinal microbial community and the soil microbial ecosystem, a relationship between both appears possible. Looking at the entire ecological system, the human body and its microbes can be regarded as an extended genome [8].

Therefore, the question ‘To what extent does a relationship between both systems exist, for example by human exposure to different soil microbiological environments?’ arises. As human activities are changing the distribution and abundance of soil microorganisms, e.g., by agricultural land use [9], the resultant changes in microbial ecosystems may not only affect biogeochemical cycles but also human health. This led us to a novel environmental microbiome concept as a potential explanation for the relationships between human health and the soil environment. In the following, we will explore the potential relationship between soil and the human intestinal microbiome.

In this context, we discuss the soil microbiome and its potential link to the (human) intestinal microbiome and assess the possible interrelation of the human intestinal microbiome and the soil microbiome.

## 2. The Complex Relationship between the Soil Microbiome and the Human Intestinal Microbiome

Since the start of the Human Microbiome Project in 2007, aiming at sequencing all microbes (eukaryotes, archaea, bacteria, viruses) inhabiting human body sites, the Human Microbiome Project has developed into a major field of biomedical research focussing mainly on the intestinal microbial community that plays a major role in human health and diseases [3,10]. The intestinal microbial community represents an ecosystem of a trillion microbial cells with an aggregate 9.9 million microbial genes across the fecal microbiome [11]. The greatest number of cells within the human gut is found in the colon which supports a diverse and dense population of microbes, dominated by anaerobes that utilize carbohydrates [12]. By comparison, the lowest number of cells found in the small intestine (Table 1) is due to properties that limit bacterial reproduction such as high levels of acids and antimicrobials [12]. Also, short transit times in the small intestine limit bacterial reproduction [13]. The colonization of the human gut starts at birth, with the rapid expansion of microbial diversity, influenced by endogenous and exogenous factors [3], such as human genetic variation as well as diet, infections, xenobiotics, and exposure to environmental microbial agents including the large plant and soil microbiome [3]. With respect to the numerous and diverse functions of the intestinal microbiome in human health, it is evident that it is also involved in numerous gastrointestinal (GI) and non-gastrointestinal diseases, such as obesity/metabolic syndrome, atherosclerosis/cardiovascular diseases, neurologic/psychiatric diseases and others [3]. It is therefore one of the most dynamic topics in biomedical research [3]. Moreover, major advances in recent years provide increasing individual diagnostics, preventive as well as therapeutic options for patients with inherited or acquired malignant or non-malignant diseases, because the individual microbial community is central for the interplay between microbes and hosts and is involved in a large number of normal biological/physiological processes [3]. All in all, it can be stated that, in recent years, the intestinal human microbiome has become one of the most dynamic areas of biomedical research and holds an enormous potential for interventions, regarding human health and disease [3].

In view of the fact that, phylogenetically, humans developed in close contact with soil as their physical basis for living, providing shelter and water as well as food for daily life, the question arises as to whether the soil microbiome as an exogenous parameter affects the development of the human intestinal microbiome. Soils existed globally a long time before mammals and hominids came into existence and are by far the most extensive natural microbial gene reservoir on earth [14].

Since 2010, the Earth Microbiome Project has focused on this gene reservoir. It is a major collaborative effort to characterize microbial life on this planet by using DNA sequencing and mass spectrometry of crowd-sourced samples to understand patterns in microbial ecology across the microbiomes and habitats of the earth [15,16].

On a per gram basis, the intestine, specifically the colon, has the highest concentration of cells of all biomes listed in Table 1 [17,18]. Considering the diversity of these habitats, the estimated number of species on a per gram basis suggests that soils harbor the most diverse populations of any environment with several thousand species (Table 1) [17]. Compared to soil, the number of species in human feces is approximately a factor of 10 lower (Table 1). However, in soils, a large proportion of cells (~80%) is inactive compared to the human gut, where only approximately ~20% of cells are dormant [19]. Considering the dormancy of cells, the total number of active species in the human gut and soil maybe comparable.

The main factors that presumably determine the human intestinal microbiome are (i) host genetics and metabolism (heritage), (ii) lifestyle (environment) in particular, and (iii) diet and nutritional habits [12,20,21].

## 3. The Human Intestinal Microbiome—Its Development and Evolution

The microbial diversity in the human gut is a coevolution between microbial communities and their hosts [30]. “Ancient” microbes evolved symbiotically or commensally with humans and are most likely beneficial rather than pathogenic [31,32].

To identify the evolutionary history of the biosphere, it is crucial to explore the microbiome of different hosts and habitats. Human-associated intestinal microbial communities are more similar to one another than to those of other mammalian species [30]. When mammals have been classified as herbivores, omnivores and carnivores, their microbiota are clustered into groups that meet these categories. However, a strong predictor of gut microbiota composition and functionality is also intestinal physiology. Gut microbiotas of herbivores are different depending on whether they were hindgut or foregut fermenters [30].

Interestingly, the gut microbiomes of several mammalian lineages have diverged at roughly the same rate over the past 75 million years [4]; Contrary to expectations, the number of dietary transitions within a lineage does not influence rates of microbiome divergence but instead, some of the most dramatic changes are associated with the loss of bacterial taxa, such as those accompanying the transition from terrestrial to marine life [4] and hence the loss of contact with soil. Another dramatic and fairly recent change has been an acceleration of divergence between humans and other primates due to the massive loss of bacteria in the human lineage [33]. Nutrition/diet was of paramount importance for the clustering pattern in primates [33]. Human gut communities seem to be comparable to those of other omnivores and are most closely related to the Bonobos or Pygmy chimpanzees, whose diets include fruits [30]. Thus, based on comparative measurements of the gut microbiota of humans and primates alone, the human species might be viewed as unspecialized frugivores, whose flexible diet includes seeds and meat depending on availability. The other hominids (Great Apes), whose diet consists primarily of plants, seem to be associated in an intermediate position between the omnivorous primates and non-primate herbivores [30]. While most insects contain only dozens of microbial species in their guts, mammalian guts contain thousands of species [5]. Herbivore guts exhibit a high bacterial diversity [5], including even plant-associated species, such as endophytes [34]. Since endophytes reside inside plant tissues, they may survive stomach digestion.

The microbial population of the human gut derived from the ancestors, individually from the mother through vertical transmission during gestation, during birth, and after birth through contact with maternal body sites, with the greatest contribution of the maternal gut [35]. Within the first three years of life, the gut bacterial phylogenetic composition evolves to an adult-like community independent of the geographic area [36]. Mothers are the source for the transmission of microbes and gut colonization during and after birth. This process is affected mainly by the way of birth (vaginal versus Caesarian section) and baby feeding (breastfeeding versus infant formula). With increasing age, the gut microbiota develop similar to other family members—due to shared households, the environment seems to be a stronger predictor than host genetics [36].

Alterations in the foraging behavior and diet of early Homo species also included interaction between family members but in a different way. In the ‘grandmother hypothesis’ [37], foraging and sharing of so-called ‘underground storage units’ (plant roots, bulbs and tubers) by older females gained importance after climate-driven changes in habitat and transformed Homo biology, ecology and societies [38]. According to this hypothesis, which is supported by isotope studies [37], older women foraging for tubers was essential for the nourishment of children, which induced greater reproductive fitness [37]. An important role of underground roots, bulbs and tubers as reported in the diet of early Homo species can still be observed in a Tanzanian population of traditional foragers inhabiting arid savannah woodlands [37]. It implicates the ingestion of soil and possible positive consequences thereof [37].

It is suggested that dietary intake has a stronger influence on gut microbial composition than host genetics [39]. The GI microbiota can even influence host genes, thereby regulating energy expenditure and storage [39]. These findings are further supported by a large scale study of the gut microbiota in over 1,000 healthy individuals, which revealed no similarity among relatives not sharing a household [40], while genetically unrelated individuals sharing a household showed significant similarity. The results suggest that host genetics play a minor role in shaping the gut microbial community with an overall microbiome heritability below 8%. Thus, gut community composition must be predominantly shaped by non-genetic factors [40] related to the environment, including lifestyle and diet.

The importance of the environment is also shown by the fact that with increasing age, the variation between individuals decreased [36]. Further, a pronounced difference in the phylogenetic composition of the gut microbiome from different regions became evident, with lowest bacterial diversity in the urban US citizens compared to rural Amerindian and Malawian populations [36]. The highest diversity of bacteria and genetic functions ever reported in a human group was found in a remote secluded population of hunter-gatherers in the Amazon jungle [41]. From the above, we conclude that short-term as well as long-term changes of the microbiome do occur on an individual level as well as on a community level and contact with soil plays a role in both scales.

## 4. Human Intestinal Microbiome and the Environment—Lifestyle

The living environment of urban dwellers shows a lower natural biodiversity and exposure to environmental microbes [42]. The loss of contact with outdoor-associated natural beneficial microbiota indirectly affects the human gut microbiome and may have negative consequences on human health [43]. Our ancestors were in close contact with soil, due to their lifestyle, i.e., practicing agriculture and animal husbandry. Research documents that children encountering early contact with environments that are less hygienic such as outdoor settings and farms are less susceptible to develop autoimmune diseases [44]. This is supported by the ’hygiene hypotheses’, which suggest that environments with rich microbial diversity protect against allergies and autoimmune disorders [45,46]. Accordingly, not only commensal microbes but also soil pathogens appear to potentially contribute to human immune tolerance by stimulating immunoregulatory pathways [47]. Nevertheless, it is important to note that modern hygiene, antibiotics, and modern agricultural practices have contributed tremendously to a major reduction in human disease burdens and mortality.

There is further evidence that soil biodiversity is interrelated with the gut microbiome. In particular, the gut microbial diversity in mice was increased by exposure to soil microbes [48]. Gut microbial diversity could increase in mice that are in contact with non-sterile soil on a normal diet, while it was unaffected by sterile soil [6]. While the addition of environmental microbes, increased gut biodiversity, a limited effect on the most abundant bacteria was described [48]. Thus microbes contribute primarily to microbial diversity while soil can change the community composition, and seemed to affect it to a similar degree as diet [6]. Results from animal studies suggest that contact with soil and its microbiome is beneficial for healthy gut microbiota [6].

The health of mammals is largely affected by domestication, which can be related to a less diverse gut microbiome observed in horse species, compared to undomesticated species [49]. This reduced animal gut diversity also holds true for most zoo animals with regard to their free living counterparts [50].

A recent study of the gut microbiome of terrestrially living baboons showed that soil is the most dominant predictor for shaping the gut microbiota with a 15 times stronger effect than host genetics [7]. While the vegetation was not strongly determining the gut microbiota, the fact that the diet of the omnivorous baboons is in close contact with soil supports the potential transmission of soil microbiota for gut colonization [7].

**Figure 1:**
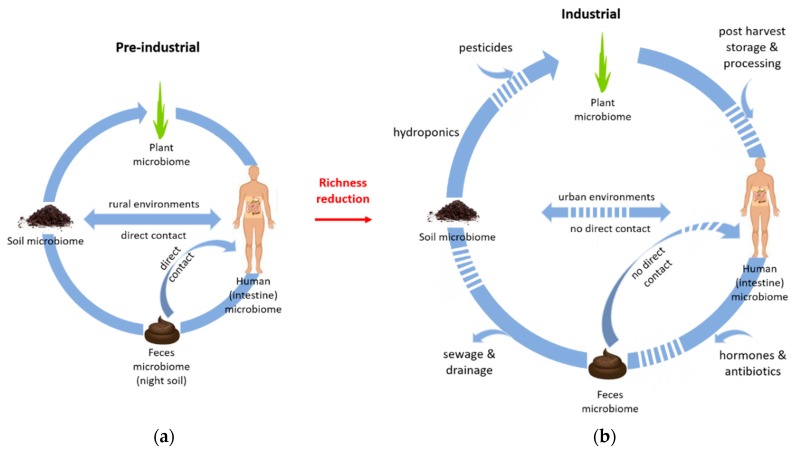
The microbiota in our environment influence the human intestine microbiome, via direct contact with soil and feces as well as via food (quality). Our ancestors lived in close contact with the environment (**a**, a cycle for pre-industrial microbiota). In contrast, human activities such as urbanization, industrialization of agriculture, and the modern lifestyle, including the use of pesticides and antibiotics as well as hormones (medication), together the loss of direct contact with soil and feces has depleted the richness of and overlapping with microbiota (**b**, a cycle for industrial microbiota). This depletion of microbial richness in all compartments can substantially affect human health.

The close link between reduced soil biodiversity (due to alkaline soil conditions) and gut microbial richness in baboons is an aspect that deserves particularly critical scrutiny in view of the global megatrend of biodiversity loss, especially for sustaining human health. Rural environments that are rich in microbiota, such as traditional farms, have been shown to have health benefits in humans [21]. In particular, manual agriculture with close contact to soil, practiced by Amish communities, in a microbial rich rural environment has shown significant beneficial effects on immune functions compared to rural Hutterites who practice mechanized agriculture [44]. However, changes in human life style can ablate the protective health impact due to changes in diet, living conditions and environmental biodiversity [21]. In pre-industrial times, small structured farms dominated and a large part of the population was working in the agricultural sector, pastoralists or hunter gatherers, and so their lifestyle was in close direct contact with nature (i.e., soil, plants, Figure 1).

Even the recycling of human feces in the form of ‘night soil’ that re-entered the agricultural sites depicts the closed cycling of resources in those times (Figure 1). Nowadays, in automated animal husbandry, there is little direct contact with feces apart from the use of manure. Manure of unmedicated livestock, e.g., in organic agriculture, may provide compensating beneficial effects by reintroducing gut microbiota into the soil microbial ecosystem.

Increasing global population and the need for housing and food have intensified agricultural practices and urbanization. Growing industrialization of agriculture results in reduced soil biodiversity [51]. Already over 50% of the world’s population lives in cities, which is expected to increase to approximately two-thirds by 2050 [52]. The ongoing global urbanization has led to a loss of contact with the natural environment by separation from the outdoors [53]. The reduced contact with microbes in the living environment, but also increased sanitation and use of antibiotics pesticides and hormones [43] depleted the richness of gut microbiota (Figure 1).

Of particular importance in this context is the fact that the richness of gut bacterial species in adults is higher in rural societies as compared to urban communities [41,54,55], while lower beta diversity (variation between individuals) was observed in rural populations [54]. The lifestyle of hunter-gatherers, which is close to our ancestors, showed the highest richness of the gut microbiome [55].

Martínez and colleagues [54] proposed that both environmental exposure and horizontal transmission of symbionts (collectively called microbial dispersal) are likely to drive the gut microbiome in rural populations, while the urban lifestyle results in the dispersal limitation of microbes. This low dispersal can explain the high inter-individual variation of the human gut microbiome of urban citizens (beta diversity). Limited dispersal combined with sanitation, medication and dietary changes reduces the successful colonization and hence gut microbiome richness [54]. A diet that is high in fibers and complex carbohydrates, which is typical for rural African populations, may preserve gut microbiome richness unlike the consumption of highly processed food that is common in urban areas of industrial western countries [56].

Hygienic measures reduce the risk for transmission of pathogens, but also of gut symbionts [54]. This limitation in dispersal via the modern lifestyle reduces the possibility for homogenization among individuals too, and leads to high beta diversity in Western populations, thus successfully linking the colonization of species by dispersion, reducing richness [54].

Thus, fecal contamination of water resources poses a significant risk for human health by spreading infectious diseases from fecal pathogens [57]. Sanitation practices such as water treatment as well as microbial hazard and risk analyses of drinking water resources are state of the art for developed Western countries and reduce the risk for infections with intestinal pathogens. The challenges in microbial source tracking for fecal contaminations are the differentiation of the origin, and ensure a target specificity for feces by excluding the identification of genetic markers that are similar but occurring in extra-intestinal habitats such as soil, plants and other environmental sources [58,59].

Besides the many ecological functions, such as the production of biomass and support of biodiversity, soil has a unique function to provide clean drinking water [60]. This soil function is enhanced by the soil biodiversity too, likely due to their involvement in, improving soil structure and water infiltration, and hence percolation through the profile. This is highly beneficial for the filter and buffer capacity of soil to ensure contaminant and pathogen removal via size exclusion, adsorption and die-off (mineralization and metabolization, [47]). This fact further stresses the importance of soil microbiota for human health, increasing the degradation of harmful pollutants, thus reducing the impact of poor anthropogenic sanitation.

Urban citizens do not only lose contact with soil but also with feces (Figure 1), e.g., because there is no need to clean stables. The importance of fecal microbiota for the development of the gut microbiome is supported by the fact that vaginally delivered infants share a higher proportion of fecal microbiota with their mothers than those delivered via Cesarean section [61]. Soil and animal feces may be important for the evolution of the human gut microbiome considering that, after weaning, infants crawl on the ground and explore the environment with their mouth [6]. A rich environment during this phase is important to human health for immunomodulatory development in early life.

As mentioned above, with protection from any form of pathogens, artificial environments such as cities can be seen as habitats that eventually share a lower number of beneficial microbes and may concentrate pathogens. Even in most cases, the exposure to feces has a great potential to perturb an otherwise healthy gut microbiome [62]. The hygiene hypothesis suggests that both communalists and pathogens can stimulate immune functions.

Access to more biodiverse areas in urban environments, such as green spaces and parks, is related to health benefits regardless of socioeconomic status [63], which can be associated with the exposure to rich environmental microbiota. Hence, recent studies on urban re-wildering to improve the urban biodiversity of our living environments can be protective against immune disease by greater contact with a diverse set of environmental microbiota and consequently improve human health [64].

Therefore, we assume that the modern human lifestyle and the loss of direct contact with soil cause interruptions in the microbiological cycle in urban environments in contrast to pre-industrial rural environments. Soil is therefore a key primary source of a healthy intestinal microbiome of humans. However, the exact way how soil and the environment shape the human gut microbiome and how lifestyle changes affect the gut microbiome needs to be further elucidated. It is a dynamic research topic with relevance for preventive medicine.

## 5. Human Intestinal Microbiome and Diet/Nutrition

Besides the urban lifestyle and loss of contact with nature, our diet has also changed within the last decennia. In order to preserve food for long transport, storage and distribution, it is often sterilized. In addition to more processed nutrition, the intake of more energy-rich food, abundant in sugars and fat, decreases the biodiversity of the intestine [65]. Often, this results in a vicious cycle as the promoted microbes in the gastrointestinal tract are under selective pressure to manipulate host eating behavior and may generate cravings for unhealthy foods that suppress their microbial competitors [65].

Medication, in particular the intake of specific drugs, mainly drives the gut microbiome of Western populations and explains the greatest total variance of the fecal microbiome as shown by a large size Danish study [66]. Increased medical antibiotic intake as well as increased meat consumption has led to an increasing number of antibiotic-resistant bacteria and genes and has caused serious environmental problems. Antibiotics do not only eliminate pathogens but also beneficial microbes inhabiting the human body, thereby dramatically changing community composition. Antibiotic resistance can be spread between bacterial populations via the horizontal transfer of antibiotic resistant genes; the gut is the habitat where this preferentially happens due to high population densities [21]. Among antibiotic resistance areas, hot spots are municipal wastewater systems that show high loads of bacteria [67]. In this context, the consumption of genetically modified plants should also be considered with caution, because the modified genes could be transferred via bacteria into the rhizosphere or the intestine [21,68].

A large scale study of fecal microbiomes including clinical and questionnaire-based covariates reported that stool consistency had the greatest effect size on the fecal microbiome, while oral medication explained the greatest total variance [66]. Among the factors that account for the combined effect on fecal microbiome composition are also dietary details such as fiber or fruit consumption and bread preference.

That diet is relevant for shaping the human gut microbiome is further supported by the study of Martinez et al. [54] which suggests that a diet rich in plant derived carbohydrates and fibers is a stronger predictor for gut microbial diversity patterns in the less developed areas of Papua New Guinea than antibiotics, although this is a locally common medication. The gut microbiome of hunter-gatherers with no access to medication [55] showed significant differences in phylum- or genus-level abundance between males and females, indicating a traditional separation of work and diet between genders. It is suggested that women are more likely to stay in one place with the family having a diet rich in tubers and fibers, while hunting was conducted by men, which shaped their gut microbiome [55].

The gatherer–hunter community in Tanzania showed a low content or total absence of Bifidobacteria in the gut microbiome [55], which usually show high abundance in infants that are breastfed [36]. Bifidobacteria are a dominant part of the infant gut community throughout the first year of life. In addition, Bifidobacteria are a vital component of gut microbiome of the Western civilization due to dietary preferences, such as dairy and meat consumption [54]. The lower proportion of Bifidobacteria compared to fiber utilizing bacteria in the human gut microbiome of vegans [54,69] supports the idea that distinct dietary habits such as the consumption of animal-derived products can affect the proportion of functionally different bacteria in the gut.

The microbiomes of non-Westernized populations resemble those of vegetarians and vegans [69,70]. The functional categorization of genes that were obtained from the gut of Amerindians with a typically protein-rich diet showed parallels to that of carnivorous mammals [36]. In contrast to the microbiomes of US citizens, higher proportions of the functional genes encoding for glutamate synthase were observed in the microbiomes of Malawians (inhabiting East Africa) and Amerindians (indigenous peoples of the Americas), who traditionally eat corn and cassava. This parallels the differences between herbivorous and carnivorous mammals [36].

In this context, it is interesting that, in a Danish study, the dietary habits that were considered as relevant for shaping the gut microbiome identified a set of carbohydrates as relevant, such as fruit, bread and alcohol consumption [66].

Specific types of food result in predictable shifts in intestinal microbiota, and hence the human intestinal microbiome can be directly affected by the diet [70]. Human diet has changed in the industrial age from a mainly seasonal and locally produced large variety of crop species to few high-yielding varieties. Together with increasing monoculture cropping, the use of pesticides (Figure 1) further reduces soil biodiversity. As soil microbes colonize the plants, soil biodiversity and plant microbiome diversity may be different before and after harvest [71].

The change in human lifestyle also includes several post-harvesting operations before consumption (Figure 1). These operations include cleaning, milling, separating, mixing, drying/hydrating, heating, dispersing, packaging, storage, distribution, transport and others. However, by sustaining soil biodiversity, in particular symbiotic microbes, food preservation measures could be reduced. Symbiotic plant microbes such as arbuscular mycorrhizal fungi have been shown to reduce storage-induced pests on staple crops such as potatoes [71]. High species richness can effectively reduce the risk for tuber disease [71]. Beside the reduction of pest risks, root symbionts can increase the nutritional quality of food/crop, including vitamins, mineral content (macro- and microelements), and antioxidants, together with other secondary plant metabolites that are beneficial for human health [71,72]. This underlines that a healthy diet and lifestyle are coupled via the consumption of food from farms which use soil management practices fostering soil biodiversity. Modern changes in farming and nutrition also include plant breeding efforts, e.g., to reduce the bitterness of Brassicacae, such as broccoli, cauliflower, and cabbage. The bitterness is due to glucosinolates, which help the plant to resist pathogens and is assumed to be an anti-cancer metabolite. Hence, the digestive function of glucosinolates is mostly depleted in the human gut [21]. This adjustment to nutrition occurs likely through the functional genes, in the same way as the transformation and degradation of drugs in the human gut.

As with bitter plant species, the consumption of fresh fruit that are almost un-processed is beneficial for human health specifically as soil biodiversity stimulates the secondary metabolite production [71]. While the ingestion of heavily processed food such as bread would provide different carbohydrate sources or fiber to the gut microbial community, the effects on secondary metabolites via high or low soil biodiversity are lost during processing [71].

In contrast to traditional smallholder farming, large scale farms, rather common in many industrialized countries, perform intensive farming practices, such as monoculture cropping of few plant species for optimizing yields. This has reduced the variety of food for humans and additionally increased the potential threats through contaminants due to the use of agrochemicals. Therefore, organically grown vegetables show a higher biodiversity of microbial endophytes and epiphytes than those conventionally grown [21]. A recent meta-study of agricultural soils has shown that organic farming is a means to enhance soil microbial abundance and activity [73].

We conclude that on top of antibiotic medication, the elimination of microbes from food via processing has direct impacts on the human gut microbiome. Anyhow, the intake of diverse food rich in fibers and secondary plant metabolites, with living microbiota, from a diverse soil environment may positively influence the gut.

## 6. Soil Microbiome as a Functional Ecosystem—Potential Links to the Gut Microbiome

Globally, soils are highly diverse, as are their microbes. There are only a few species that can be found in all soils, while there are numerous rare species that only occur in particular soils or geographical areas [47]. This enormous heterogeneity of soil biodiversity and its relation to geographical areas was addressed by several research groups [74,75,76] and a global soil biodiversity atlas and homepage was elaborated (https://www.globalsoilbiodiversity.org/atlas-introduction/). Thus, it has become possible to compare the geographical data of soil microbiomes with human gut microbiomes [43].

Unexpectedly, Tasnim et al. [43] reported that there was little overlap between soil and gut microbes at lower taxonomic levels. Human fecal samples were dominated by Bacteriodetes and Firmicutes phyla, whereas soil samples were dominated by Proteobacteria and Verrucomicrobia. While there is still a lack of data for comparing the gut and soil microbiome over large geographical areas and a number of critical methodological issues, there seem to be fundamental differences between the two habitats. One major difference is that soil is a medium limited in carbon and energy and microbes maintain a starving-survival lifestyle [77], whereas, in the human intestine, carbon and energy are abundant. The situation is different in the rhizosphere, where a constant proliferation of C-rich root exudates exists, providing nutrients and energy. The microbial community of the rhizosphere is related to the root endophyte community as well as the bulk soil community [78]. Indeed, the rhizosphere microbiome of the red clover (a legume: the plant family with highest root exudation) harbors Bacteriodetes as well as Verrucomicrobia plus their symbiotic root nodule bacteria [79]. Internal and external symbionts at plant roots differ, with the former like bacteria in animal guts while the latter more akin to those colonizing the skin [80].

Beside phylogenetic similarities between the plant rhizosphere and the human gut microbiome, there are many functional similarities [81]: (i) The gut and rhizosphere are open systems with large surface areas overpopulated with microbes. (ii) The gut and rhizosphere are characterized by a gradient of oxygen, water and pH, resulting in a diversity of niches. (iii) The gut and rhizosphere microbiome structures are shaped according to host genotype and age. Both provide protection against pathogens and modulate the immune system [81]. These cross-kingdom similarities in microbiome ecology lead to the discussion of similar strategies for the biocontrol of pathogens in plants and in humans [82]. One commonly observed phenomenon is that the survival rates of invaders are inversely related to the diversity of the native microbiome. This can be explained by higher resource uptake and a consequent reduction in niche availability [83]. These findings point toward the paramount importance of sustaining rich diversity in soils/rhizospheres in the first place in order to avoid the necessity for biotechnological interventions at a later stage (e.g., introduction of probiotics or symbionts as discussed in [84]).

In order to sustain rich rhizosphere biodiversity, we have to understand the major drivers of this functional ecosystem: The rhizosphere microbiome is related to soil type, moisture, age, plant genotype and root lysates and exudates [5]. Similar to the human gut, the rhizosphere offers a vast surface (via root hairs, or microvilli [5]). In addition, both systems have a considerable heterogeneity in common, and hence multiple microenvironments to be colonized by a large number of species compared to other environmental habitats such as air, leaf surfaces, or water bodies [18,24]. These microenvironments are reduced if there is extensive soil tillage or in hydroponic cultivation. The rhizosphere microbiota are considered to be enriched in r-selected species likely due to high nutrient availability. In contrast to that, the bulk soil generally shows a more stable population of slower growing microbiota, such as Acidobacteria, Chloroflexi, Verrucomicrobia, and Planctomycetes, which have been previously described as soil oligotrophs [85,86]. They can survive extended periods of starvation [77] and are essential for the re-inoculation of new seedlings. The application of excessive doses of mineral fertilizers reduces survival conditions for soil oligotrophs [87]. Besides improper land management, land use change has serious effects on microbial communities. In particular, soil sealing and soil erosion lead to the loss of large areas of soils together with their typical indigenous microbial populations, a loss which cannot be reverted [88].

Also biofilms can be found in both the human GI tract/gut [89,90] and the rhizosphere [91,92]. The gut and soil rhizosphere are nutrient-rich environments and follow circadian cycles, such as the fixation of nitrogen in rice roots, which is higher during daytime than in the dark, as well as the hormone melatonin in the human GI tract, which controls the biological clock [5].

The gut as well as the rhizosphere microbiome can be considered as “superorganisms” contained in/around the host, which are of paramount importance for the health and performance of the host, because (i) the gut microbiota are important for producing essential amino acids and vitamins such as B12 and K, and (ii) the root microbiota for producing hormones that are promoting plant health by improved nutrient acquisition, resistance to abiotic (i.e., drought) and biotic (i.e., pathogens) stress, and by sustaining growth [21].

The deficiency of some micronutrients in humans, derived from nutrient-depleted soils, can have substantial effects as co-factors in metabolism, modulating enzyme activities, or functioning as coenzymes [93]. Any reduction of aboveground and belowground soil biodiversity threatens soil ecosystem functions [94]. These losses in soil biodiversity are due to direct anthropogenic activities [47]. Indirect anthropogenic effects due to climate change will increasingly impact soil functioning through stressing soil organisms and affecting biodiversity, as indicated by soil warming experiments [95,96,97].

Worldwide urbanization as well as the mechanization of agriculture have dramatically increased during the last century. In combination with the use of agrochemicals such as mineral fertilizers and pesticides, soil biodiversity is reduced. In human medication too, a strong transformation occurred during this time by the use of antibiotics and hormones. The substantial effect of medication on the gut microbiome of the Western population was recently proven [66].

From the above-mentioned structural and functional similarities between the soil rhizosphere and the human gut microbiome, we conclude that both can be considered as functional ecosystems, which interact which each other. This interaction has been decreasing in recent times, potentially reinforcing losses of biodiversity, which have occurred in both systems.

## 7. Summary

Recent research data indicate that the modern lifestyle/environment is the most active driver in shaping the human intestinal microbiome despite the confounding influence of dietary habits, culture, and host genetics. The soil (rhizosphere) microbiota clearly influence the quality and storage of our food apart from the impact of post-harvest processing. In this context, more research is necessary to demonstrate how biodiversity of beneficial microbes in our food can be preserved. Furthermore, specific ways of agricultural practices, especially soil management, may improve the current food quality.

Basically, recent findings suggest the investigation of the soil and root microbiota in more detail may identify effects on human health, possibly, among others, by adopting a lifestyle of former generations. This lifestyle, such as the reduced consumption of livestock and dairy products and the intake of a higher diversity of nutritional fibers and bitter substances may have beneficial effects on our health. The intake of mostly unprocessed organically grown regional products is one way towards this goal. Further, wild relatives of the currently used high-yielding crop varieties could increase aboveground and belowground biodiversity, and hence provide benefits to soil and human health, e.g., through reintroducing lost beneficial microbes.

A rich soil microbiome would also have several advantages for the terrestrial ecosystem through increased nutrient use efficiency and uptake, which may improve plant yields as well as plant resistance and resilience against global climatic change and biotic stressors. The fact that the gut microbiome of hunter-gatherers has a higher species richness than that of humans that are nourished by Westernized food argues for agricultural practices that promote sustainable soil use and human health. Regarding food security under the aspect of predicted changes in human demographics and environmental change, it is of paramount importance to ensure the biologically sustainable use of land and soil.

## 8. Conclusions and Outlook

The soil contributes to the human gut microbiome—it was essential in the evolution of the human gut microbiome and it is a major inoculant and provider of beneficial gut microorganisms. In particular, there are functional similarities between the soil rhizosphere and the human intestine. In recent decades, however, contact with soil has largely been reduced, which together with a modern lifestyle and nutrition has led to the depletion of the gut microbiome with adverse effects to human health. Therefore, we suggest increasing research on the geographical and functional relationships to identify the causes and effects between soils and gut microbiota in order to benefit human health and the environment.

## Figures and Tables

**Table 1:** Number of microbial cells contained in environmental/human samples such as soil, sewage and human intestine.

Habitat	Number of Cells per g	Number of Cells per mL	Species Diversity
Soil	10^7^–10^9^ [22]	10^10^ [23]	4 × 10^3^–5 × 10^4^ species per g soil [22]
Sewage		10^9^ [24,25]	25 per mL [24]
Marine water		10^5^–10^6^ [18]	
Air		1 (=10^6^ cells/m³) [17]	
Human gut	10^12^ [26]		4 × 10^2^ species per g feces [27]
	10^11^ [28]		5 × 10^3^ species per g feces [18]
Colon (large intestine)		10^11^ [29] 10^11^–10^12^ [30]	
Ileum (lower small intestine)		10^8^ [29]	
Duodenum and jejunum (upper small intestine)		10^3^–10^4^ [29]	
Human mouth (saliva)		10^8^ [18]	
